# 3D Simulation Modeling of the Tooth Wear Process

**DOI:** 10.1371/journal.pone.0134807

**Published:** 2015-08-04

**Authors:** Ning Dai, Jian Hu, Hao Liu

**Affiliations:** 1 College of Mechanical & Electrical Engineering, Nanjing University of Aeronautics & Astronautics, Nanjing, Jiangsu, P.R. China; 2 Institution of Stomatology, Nanjing Medical University, Nanjing, Jiangsu, P.R. China; Monash University, AUSTRALIA

## Abstract

Severe tooth wear is the most common non-caries dental disease, and it can seriously affect oral health. Studying the tooth wear process is time-consuming and difficult, and technological tools are frequently lacking. This paper presents a novel method of digital simulation modeling that represents a new way to study tooth wear. First, a feature extraction algorithm is used to obtain anatomical feature points of the tooth without attrition. Second, after the alignment of non-attrition areas, the initial homogeneous surface is generated by means of the RBF (Radial Basic Function) implicit surface and then deformed to the final homogeneous by the contraction and bounding algorithm. Finally, the method of bilinear interpolation based on Laplacian coordinates between tooth with attrition and without attrition is used to inversely reconstruct the sequence of changes of the 3D tooth morphology during gradual tooth wear process. This method can also be used to generate a process simulation of nonlinear tooth wear by means of fitting an attrition curve to the statistical data of attrition index in a certain region. The effectiveness and efficiency of the attrition simulation algorithm are verified through experimental simulation.

## Introduction

Tooth wear refers to losses of enamel and dentin caused by direct contact between opposing teeth or between teeth and foreign objects, such as food [1]. It is an irreversible process that occurs continuously and can run parallel to tooth development (from eruption to the loss of teeth). Tooth wear may cause pathological changes in the dental pulp, e.g., dentin hypersensitivity and dental pulp inflammation; it may also induce decreases in vertical distance in occlusal direction, temporomandibular joint disorders, and poor masticatory function [2]. The study of the prevalence and features of tooth wear may provide an accurate basis for clinical prevention and therapy, as well as valuable information for archaeology and forensic identification [3].

So far, the study of tooth wear primarily focuses on observation and description for assessment [4]. Qualitative description usually uses classifications and ratings for the recognition of attrition or the attrition index. Smith et al. [5] proposed an 8-level classification method that can visually represent tooth wear through images, though with strong subjectivity and perhaps low precision. Quantitative measurement methods have always been a popular topic of study among researchers. Analysis of direct image measurement of the height of a cusp, proposed by Hove et al. [6], and quantitative measurement using scanning electron microscopy, proposed by Teaford et al. [7], have limited function and can only measure partial aspects of tooth wear. 3D tooth model digitizing and processing technology is a novel means for the study of tooth wear. DeLong et al. [8] arrived at the conclusion that sequential 3D tooth model comparison is the most accurate method for measuring tooth wear. Mitchell et al. [9] described an erosion detection system to detect minute levels of tooth erosion and investigated the linear interpolation error of dental erosion measurement [10]. Zou et al. [11] quantitatively measured the attrition value and the distribution area between tooth surfaces by a registering algorithm based on 3D teeth model. Rodrigueza et al. [12] proposed that high measurement accuracy and consistency can be achieved by adopting a non-contact 3D laser scanner and surface matching software. Evans [13] presented a new analysis method to predict the effects of wear using 3D digital molar modelling to relate dental parameters of size and shape. This analysis method provides a valuable reference for research of human tooth wear. The above studies can provide gradual quantitative analysis and discussion on the accuracy, consistency and distribution of tooth wear in certain phases. However, tooth wear is a slow and long-term dynamic process [7]. Learning how to intensively analyze the tooth wear process will help further our understanding of the contribution of attrition and allow us to predict the tendency of tooth wear. Accurate measurement of the attrition process is time-consuming and difficult. Relevant studies face great challenges in cost and time. Computer simulation offers a new experimental method for studying tooth wear.

Geometrically speaking, tooth wear is the physical process of consistent local deformation of occlusal contact areas. Some methods of surface deformation have been proposed for the simulation of tooth morphology transformation as follows: Parent et al. [14] proposed a method that constructs vertex, edge, and surface structures using a subdivision scheme to implement the gradual process simulation of spherical objects. Lerios et al. [15] proposed a volume-based 3D morphing method that can realize gradual changes in the 3D morphology of two different objects, but with extensive calculations. Kanai et al. [16] proposed an algorithm that establishes a corresponding feature relationship based on a harmonic map, thus accomplishing a gradual change between complex 3D models. With this algorithm, the problem of feature correspondence in morphing technology is solved, but it is still not robust enough for numeric calculation. Yan et al. [17] proposed a 3D morphing algorithm based on strain field interpolation. This algorithm is required to solve a nonlinear equation, so it involves complex calculations. Athanasiadis et al. [18] proposed a 3D morphing simulation algorithm with a random zero genus grid model based on features, which is extremely complex and difficult to generalize.

To solve the problems of tooth wear process simulation mentioned above, this paper presents a novel algorithm for the simulation of the tooth wear process; the algorithm’s feasibility has been verified through a series of simulation experiments. Section 2.1 outlines the basic procedure of simulation modeling of the tooth wear process. Section 2.2 describes the method of identification of the attrition features of occlusal surfaces. Section 2.3 describes the establishment of homogeneous attrition surfaces by feature alignment, implicit surface approximation and the strategy of contraction and bounding. Section 2.4 proposes a method based on interpolation of Laplacian coordinates to inversely reconstruct a digital simulation of the tooth wear process. Section 3 (Results) and 4 (Discussion) detail the analysis and results of this method. The experiment’s conclusion is stated in Section 5 (Conclusions).

## Materials and Methods

### 2.1 Ethics statement

All participants enrolled and the experimental procedures in this study are in accordance with the Declaration of Helsinki (revised in Edinburgh 2000). All subjects signed an informed consent form to participate in a protocol that was approved by the Ethics Committee of Affiliated Hospital of Stomatology, Nanjing Medical University, China. (No.PJ2012-027-001. Date: 20/5/2012)

### 2.2 Basic procedures

**Fig 1:**
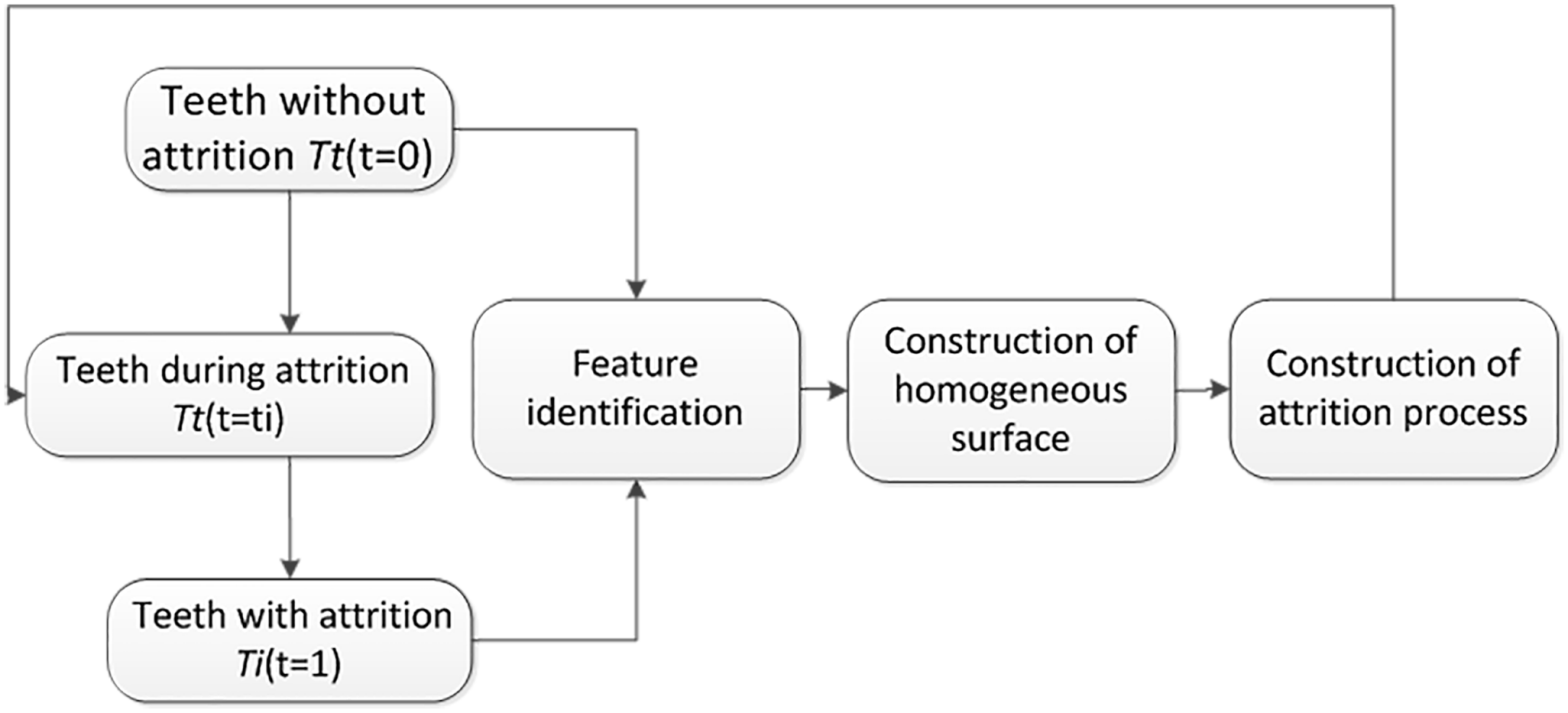
Simulation of the tooth wear process includes four stages: data acquisition, feature identification, construction of homogeneous surface and generation of attrition process model.

**Fig 2:**
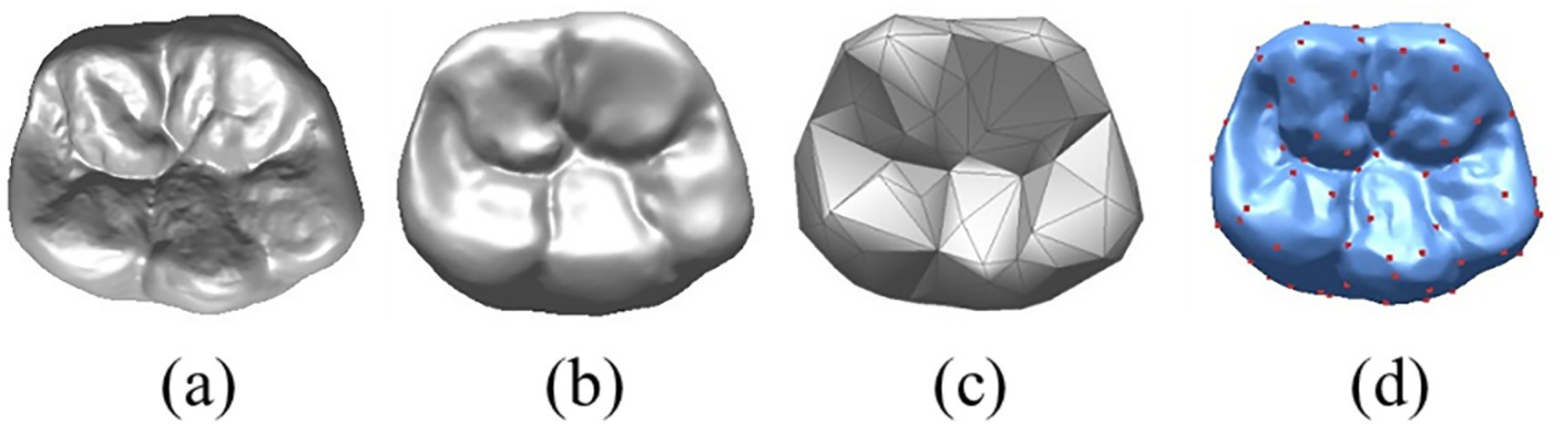
Feature points of tooth morphology. a. lower molar model [point 16594/Δ32894]; b. attrition model of first molar [point 14491/Δ28738]; c. simplified feature model [point 152/Δ212]; d. feature points marked [point 152/Δ212].

Simulation of the tooth wear process includes (Fig 1): (1) Data acquisition of teeth with attrition. To acquire surface data, a 3D optical scanner(D700,3Shape Company, Denmark) was used to scan the teeth before and after attrition (referring to Yip et al. [19] and their design of molars on the attrition level) as shown in Fig 2(a) and 2(b). (2) Identification of feature points of the tooth’s primary anatomical geometry. Efficient calculation of the matching feature points of teeth without attrition was performed using a simplified mesh method based on QEM (Quadric Error Measurement) [20]. (3) Establishing the feature matching relationship before and after attrition. A distance field function was constructed by using implicit surface interpolation, establishing the correspondence relationship between feature points, and spreading this relationship to all mesh vertices. (4) Using simulation modeling for tooth wear. Local detail features are retained using Laplacian coordinates and interpolation morphing surfaces from teeth before and after attrition are constructed, to accomplish the visual geometric process simulation of tooth wear.

### 2.3 Feature identification of attrition

While tooth shape changes through wear due to the loss of dental material, tooth wear can be modelled as a consistent morphing process. The development of the attrition process is reflected in the changes in shape of worn areas. Establishing the corresponding relationship of tooth anatomical features before and after tooth attrition is important for constructing a process simulation.

#### 2.3.1 Model presentation

Human teeth have highly detailed features such as grooves, fossae, ridges and cusps as a result of their long history of evolution. A high quality triangle mesh model can be obtained using a high-precision optical scanner. The triangle mesh model *M* can be expressed as *M* = (*V*,*K*), in which *V* = {*v*_1_,*v*_2_,…,*v*_*i*_}, *v*_*i*_∈*R* ^3^ represents the geometric information of a triangle mesh model, i.e., the geometric position of vertices set in 3D space; and *K* represents the topological information of the mesh, showing the connection mode between vertices.

#### 2.3.2 Identification of tooth wear features

Tooth wear features include anatomical features distributed on the occlusal surfaces of teeth in various shapes and sizes, such as grooves, cusps and ridges, etc., which include numerous variations among people. The positions and shape of tooth grooves are relatively constant and are closely related to the shape of the cusps. The cusps are connected along a marginal ridge around the edge of the occlusal surface. Tooth wear features can be analyzed and extracted in terms of regularity. Schroeder et al. [21] proposed a gradual mesh simplification feature extraction algorithm based on the extraction of key surface features by iteratively deleting vertices to satisfy the precision standard, such as the minimal distance value, etc. The algorithm is fast, but approximation errors occur and cannot be avoided. Lee et al. [22] proposed that mesh saliency can be used for surface feature extraction, which is quick and accurate, but the feature points extracted lack actual physical meanings. By adopting a QEM-based mesh feature extraction algorithm and calculating the overall QEM value, the mesh is quickly simplified to get the mesh surface feature model. Fig 2(c) shows the simplified feature model with mesh. Each point in the simplified model is a feature point of the initial molar model. Feature points (152 red dots) could be obtained from the initial tooth model by using a feature extraction algorithm. Fig 2(d) shows feature points on a tooth model without attrition. By observing the distribution of feature points, the feature points calculated by using this method can effectively describe the tooth’s anatomical geometry.

### 2.4 Construction of a Homogeneous Surface

Attrition occurs on tooth occlusal surfaces under the long-term action of periodic masticatory forces. The shape of the occlusal surface will change accordingly. The precondition of tooth wear simulation is the analysis of morphological changes of the tooth occlusal surface and the construction of a homogeneous tooth wear surface. In this paper, the current status of the tooth occlusal surface is defined as *St*,*t*∈[0,1]. If *t* = 0, *S*_0_ is the surface before attrition; if *t* = 1, *S*_1_ is the surface after attrition.

#### 2.4.1 Feature alignment

**Fig 3:**
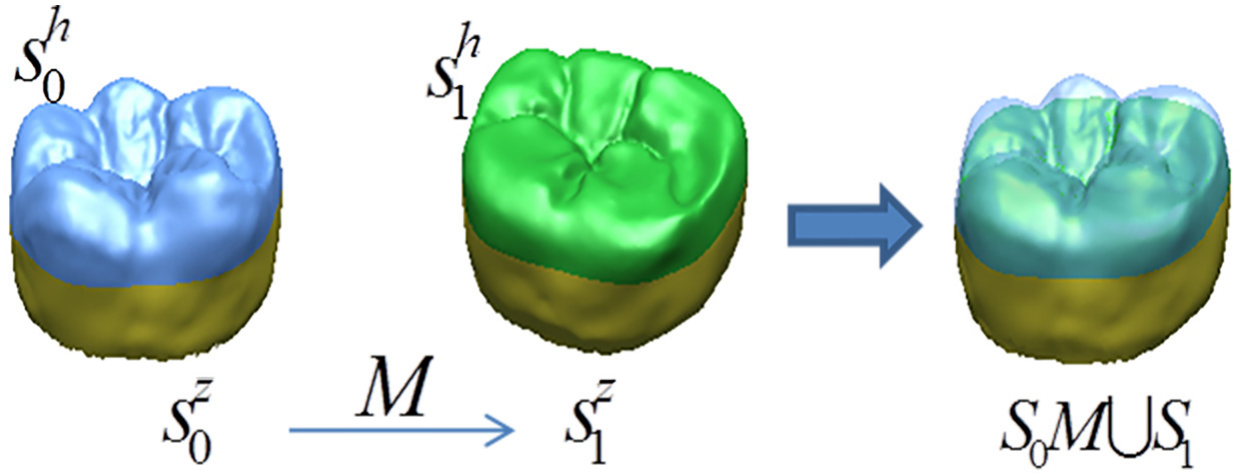
Feature alignment before and after attrition. The yellow area is a selective alignment area, the blue area is occlusal surface $S_{0}^{h}$ before attrition and the green area is occlusal surface $S_{1}^{h}$ after attrition.

Tooth occlusal surfaces before and after attrition were measured with a 3D optical scanner. Before investigating the morphological changes of the attrition surface, anatomical features, such as grooves, fossae, cusps and ridges, tooth occlusal surfaces before and after attrition must be aligned. Attrition primarily occurs on occlusal surfaces, so the model can be divided into two parts: occlusal surface $S_{t}^{h}$ and axial surface $S_{t}^{z}$, where $S_{t}\;=\;S_{t}^{h}+S_{t}^{z}$. In this paper, a selective area alignment is adopted to register $S_{1}^{z}\;=\;S_{0}^{z}M$ (*M* is a transformational matrix) for the non-changed areas before and after attrition (Fig 3—the yellow area $S_{0}^{z}$ in *S*_0_ and the yellow area $S_{1}^{z}$ in *S*_1_) by using the ICP (Iterative Closest Point) algorithm. Therefore, *S*_0_ *M* will be aligned with *S*_1_ to attain the feature alignment of the two models.

#### 2.4.2 Construction of a homogeneous wear surface

Under the action of periodic masticatory force, attrition on the occlusal surface causes *S*_0_ → *S*_1_. However, *S*_1_ is not caused by direct attrition on surface *S*_0_, but is only the surface after attrition. At this point, we must construct the homogeneous surface *S*_*h*_ generated by deformation of surface *S*_0_ to allow *S*_*h*_ ≅ *S*_1_. The homogeneous surface *S*_*h*_ can be directly constructed on *S*_1_ after feature alignment using the method of closest projection points. Due to the extensive morphological differences between *S*_0_ and *S*_1_, a large number of self-intersected data points are generated on $S_{1}^{'}$, which will influence the truthfulness and accuracy of the model. This paper presents a method that constructs the high quality homogeneous surface *S*_*h*_ by adopting a contraction and bounding algorithm after the construction of the initial surface *S*_*h*_ using the RBF (Radial Basis Function) implicit surface.

**Fig 4:**
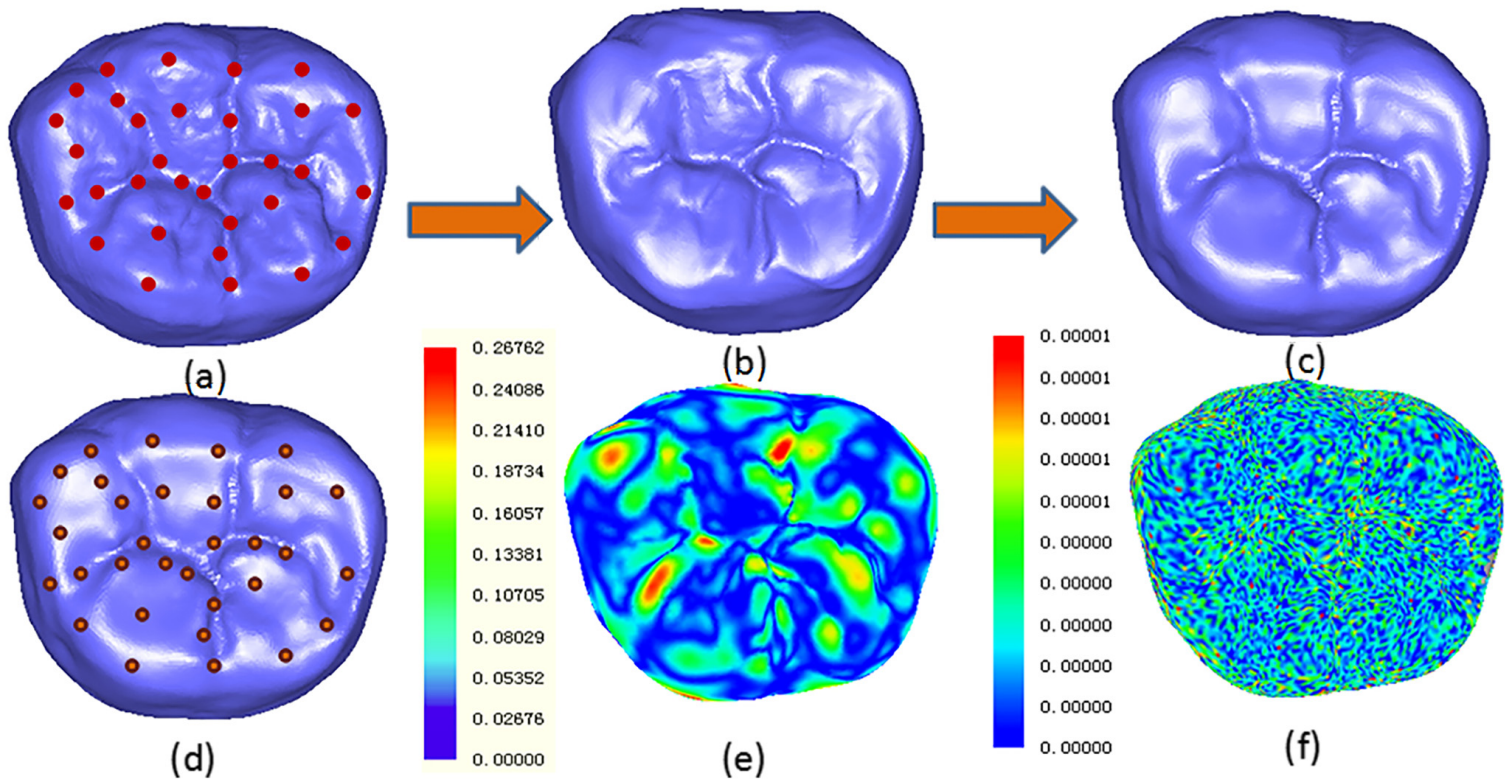
Construction of the homogeneous attrition surface. a. surface without attrition *S*_0_ [16594 points/32894 triangles] and feature points set; b. initial homogeneous surface *S*_*h*_; c. homogeneous attrition surface after three iterations of contraction and bounding; d. attrition surface *S*_1_ and feature points set; e. error distribution between *S*_*h*_ and *S*_1_; f. error distribution between *S*_1_ and homogeneous attrition surface after three iterations of contraction and bounding.

First, the feature points set {V_1_, V_2_ …, V_*n*_} on surface S_0_ is generated using the method mentioned in section 2.2, after which the feature points set of S_0_ will be projected onto the attrition surface S_1_ using the method of point projection along the normal vector to obtain the feature points set {$V_{1}^{‘},V_{2}^{‘}\ldots ,V_{n}^{‘}$}, as shown in Fig 4(a) and 4(d). Then, the distance field of feature point pairs is interpolated using the RBF implicit surface.

$$D_{i}=\overset{n}{\underset{i,j=1}{\sum }}\lambda_{j}\phi \left(\left|V_{i}-V_{j}\right|\right)+C\left(V_{i}\right)$$ (1)

In this Eq (1), *D*_*i*_ is the distance of the point pair {*V*_*i*_, *V*_*j*_} from the ith group, *λ*_*j*_ is the weight of the radial function, and ϕ(*x*) is the radial function (for 3D space interpolation, generally ϕ(*x*) = *x* ^3^). C(*x*) is a first-order polynomial, used to ensure the affinity invariability of the final interpolation surface to given discrete constraint points; for a random point V = {*V* ^*x*^, *V* ^*y*^, *V* ^*z*^}, P(*x*) is defined as:

$$C\left(V\right)=c_{0}+c_{1}V^{x}+c_{2}V^{y}+c_{3}V^{z}$$ (2)

The linear system above is solved for an unknown number *n*+4 (*λ*_*i*_ and p_0_, p_1_, p_2_, p_3_), with another four orthogonality conditions that must be supplied for a complete solution [23].

$$\overset{n}{\underset{j=1}{\sum }}\lambda_{j}=\overset{n}{\underset{j=1}{\sum }}\lambda_{j}V_{j}^{x}=\overset{n}{\underset{j=1}{\sum }}\lambda_{j}V_{j}^{y}=\overset{n}{\underset{j=1}{\sum }}\lambda_{j}V_{j}^{z}=0$$ (3)

A matrix form can be obtained by substituting (2), (3) into (1):

[APPT0][λC]=[D0] (4)

in which *A*_*ij*_ = *ϕ*(|*V*_*i*_ − *V*_*j*_|), $\text{P}=\left[\begin{array}{cc} \begin{array}{cc} 1 & V_{1}^{x}\\ 1 & V_{2}^{x} \end{array} & \begin{array}{cc} V_{1}^{y} & V_{1}^{z}\\ V_{2}^{y} & V_{2}^{z} \end{array}\\ \begin{array}{cc} \vdots & \vdots \\ 1 & V_{n}^{x} \end{array} & \begin{array}{cc} \vdots & \vdots \\ V_{n}^{y} & V_{n}^{z} \end{array} \end{array}\right]$

The initial homogeneous surface S_h_ generated by the RBF implicit surface can only accept precise interpolation of a feature points set, as there are fewer feature points than mesh vertices in the model. Fig 4(b) shows the initial homogeneous attrition surface S_h_, constructed by the distance field function of the feature points pair corresponding to interpolation, as shown in Fig 4(a) and 4(b). The error distribution between S_h_ and the final attrition surface S_1_ is shown in Fig 4(e).

There is a certain margin of error, which will influence the accuracy of the process simulation, between the initial homogeneous attrition surface obtained by the calculations above and the final attrition surface S_1_. In this paper, the strategy of contraction and bounding is adopted for iterative deformations that gradually converge to the attrition surface S_1_. First, the normal vector of each mesh vertex *P*_*i*_ on S_h_ is estimated; second, the projection point *Q*_*i*_ on S_1_ with a mesh vertex along the normal vector is calculated, where λ is a contraction factor; to decrease the self-intersection caused by contraction as much as possible, generally λ = 0.4.

$$P_{i}^{\text{'}}=P_{i}+\lambda \left(Q_{i}-P_{i}\right),\;\lambda \in \left[0,1\right]$$ (5)

Finally, weighted smoothing to a 1-ring neighborhood in mesh is performed for $S_{h}^{\left(k\right)}$, where *k* represents the current iteration time point. These steps are repeated until the error function E<*ϵ*, where ϵ = 0.001, and *m* is the number of mesh vertices.

$$E=\overset{m}{\underset{i=0}{\sum }}\left|P_{i}-Q_{i}\right|$$ (6)

After three iterations, the homogeneous attrition surface $S_{h}^{\left(3\right)}$ largely approximates the attrition surface S_1_, and the average error is less than 0.00001, as shown in Fig 4(c) and 4(f).

### 2.5 Modeling of the tooth wear process

As the substitute of attrition surface S_1_, the homogeneous attrition surface S_h_ has the same number of points and a one-to-one correspondence with the non-attrition surface S_0_. Linear interpolation can be used for the generation of in-between frames of model simulation. Linear interpolation has a better morphing effect for two objects with similar shapes. However, severe anamorphoses, such as shape distortion and contraction, will occur for large partial shape differences. Based on the description of Laplacian coordinates [24] for which the partial geometric detail of the mesh surface is translation invariant, detailed features of the model can be effectively retained before and after deformation. The in-between frame models of the attrition morphing simulation are generated by bilinear interpolation in S_1_ and S_h_ after matching their Laplacian coordinate spaces.

#### 2.5.1 Laplacian coordinate transformations

Define the triangle mesh surface M = {*V*,*E*,*F*}, in which *V* is a set of points, *E* is a set of edges, and *F* is a set of faces. The Laplacian coordinate form of vertex v_i_ is:

$$\;L\left(v_{i}\right)=\underset{j\in N\left(i\right)}{\sum }\omega_{ij}\left(v_{i}-v_{j}\right)$$ (7)

Here, L is the Laplace Operator, *N*(*i*) = {*j*|{*i*,*j*}∈E} and is a set of adjacent vertices of vertex *v*_i_, and *d*_*i*_ = |*N*(*i*)| represents the degree of *v*_*i*,_, *ω*_*ij*_ showing the weight relation between vertex *v*_*i*_ and *v*_*j*_ that satisfies the quantitative relation ∑_j∈N(i)_ *ω*_*ij*_ = 1, generally taking the coordinate of average weight *ω*_*ij*_ = 1/*d*_*i*_ to show the difference in the center of mass on a graph of points on the mesh and their adjacent vertices. As shown by the Laplacian coordinate system, the anatomical features of tooth surfaces can be well retained during morphological operations.

#### 2.5.2 In-between frame attrition model reconstruction of bilinear interpolations

δ_s_(P_i_) represents the Laplacian coordinate of vertex *P*_*i*_ on the non-attrition surface S_0_, and δ_T_(*P*_i_) represents the Laplacian coordinate of *P*_*i*_ as the corresponding point on homogeneous attrition surface S_h_. When transforming S_0_ and S_h_ into Laplacian coordinate space, the Laplacian coordinate δ_M_(*P*_*i*_) of intermediate state P_i_ during the morphing process can be acquired as follows:

$$\delta_{M}^{U}\left(P_{i}\right)=\left(1-u\right)\frac{\delta_{s}\left(P_{i}\right)}{\left|\delta_{s}\left(P_{i}\right)\right|}+u\frac{\delta_{T}\left(P_{i}\right)}{\left|\delta_{T}\left(P_{i}\right)\right|}\;\;\left(0\leq u\leq 1\right)$$ (8)

$$\left|\delta_{M}\left(P_{i}\right)\left|=\left(1-u\right)\right|\delta_{s}\left(P_{i}\right)\left|+u\right|\delta_{T}\left(P_{i}\right)\right|\;\;\left(0\leq u\leq 1\right)$$ (9)

$$\delta_{M}\left(P_{i}\right)=\left|\delta_{M}\left(P_{i}\right)\right|\delta_{M}^{U}\left(P_{i}\right)$$ (10)

It can be seen in the solution formula for δ_M_(P_i_) that the Laplacian bilinear interpolation is direct and the magnitude of the Laplacian coordinate vector quantity is interpolated simultaneously. This method can effectively avoid contraction.

When δ_M_(P_i_) is acquired, the relevant world coordinate can be solved in reverse. For this purpose, a large-scale sparse linear equation shall be solved, as follows:

$$\left[\text{L}\right]{\left[V\right]}_{M}={\left[D\right]}_{M}$$ (11)

Here, [D]_M_ is the Laplacian coordinate matrix, [L] is the Laplacian matrix of the mesh, and [V]_M_ is a world coordinate matrix. The sparse linear equation is solved using the Taucs library [25].

## Results

A plaster model of the lower first molar from a randomly chosen youth present in our clinic during the simulation experiment was used as the example of a tooth without attrition in our experiment. The tooth plaster model without attrition and the designed tooth plaster model with attrition were scanned using a Denmark 3-Shape D700, and recorded as S_0_. The scanning precision is ±0.02 mm, as shown in Fig 2(a). The computer used for the experiment is an Intel i5-3450, Memory 2GB, Win7; the simulation algorithm is developed using VC2008 and a graphic display with OpenGL2.0.

### 3.1 Attrition Simulation Experiment

#### 3.1.1 Simulation process of attrition

**Fig 5:**
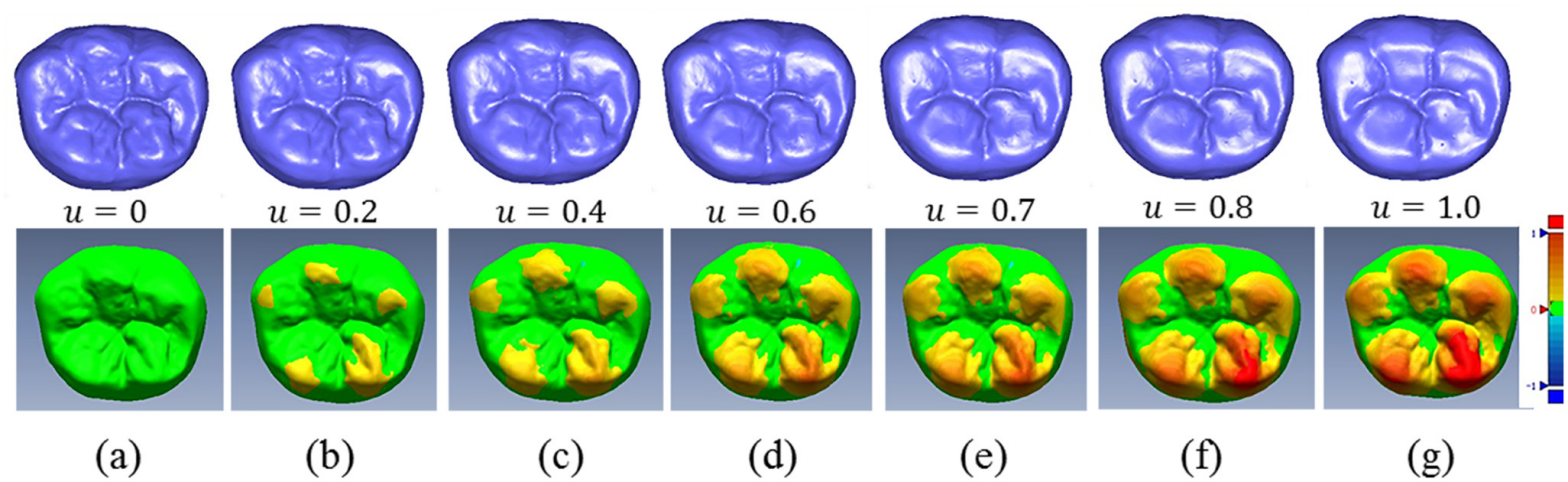
Linear attrition process simulation for the mandibular first molar attrition. a. tooth surface before attrition; b-f. tooth wear surface with parameter *u* from 0.2 to 0.8; g. tooth wear surface with parameter *u* = 1.0. The color from yellow to red shows the distribution area and degree of wear.

The tooth model *S*_1_ with attrition index between 3 and 4 was handmade with reference to the attrition morphology of Yip et al. [19]. The homogeneous surface *S*_*h*_ of tooth with attrition *S*_1_ was established using the method described in section 2.3.2, as shown in Fig 5(g). The simulation sequence of tooth wear was calculated according to the linear interpolation factor *u* under Laplacian coordinate space, as shown in the upper portion of Fig 5(a)–5(g). The lower portion of Fig 5(a)—5(g) shows the distribution area and degree of wear, along with the change in the linear interpolation factor *u*.

#### 3.1.2 Efficiency of attrition morphology simulation

The time required for attrition morphology simulation is shown in Table 1. When using QEM, the time required to extract feature points includes the time required to calculate weight values and fold overall edges. The mapping time includes solving implicit surface factors and projection smoothing operator with multiple iterations. The time required for Laplacian bilinear interpolation mainly includes solving the linear matrix. The overall computation speed required for fast attrition simulation was achieved.

**Table 1:** Simulation time of tooth wear.

	The number of points /The number of triangles	Extraction of feature points (s)	Mapping(s)	Laplacian bilinear interpolation (s)
**Tooth wear simulation**	Intact tooth(16594/32894) /Worn tooth(14491/28738)	0.91	4.22	0.72

### 3.2 Simulation Experiment on Nonlinear Attrition

The wear of a natural tooth will intensify with age due to the breaching of enamel with high hardness into dentin with slightly lower hardness. From observations of the enamel microstructure, the enamel is generally arranged in a fan-shaped distribution [26–27] from the inside to outside, so it will lead to nonlinear characteristics according to anisotropic microstructure of tooth. In this paper, the nonlinear attrition simulation is further realized by defining the DCF (Dynamic Control Function) with *u*(*t*).

#### 3.2.1 Statistics of nonlinear attrition

Currently, due to unknown tooth attrition mechanisms, the determination of DCF for tooth wear is based on data of tooth wear reported by Liu [28] in 2007. Assuming that the first permanent molar erupts at age 6 to 8, the attrition index is 0. The life expectancy used is 78 and the attrition index used is 4. The intermediate value of the age group is used for the corresponding attrition index. The data investigated by Liu [28] are shown in Table 2.

**Table 2:** Average Attrition Index of lower molar at Different Ages.

**Age *x*(year)**	8	23	30	40	50	60	66	78
**Attrition Index *y***	0	0.88	1.23	1.43	1.74	1.99	2.11	4

#### 3.2.2 Dynamic distribution curve of attrition

**Fig 6:**
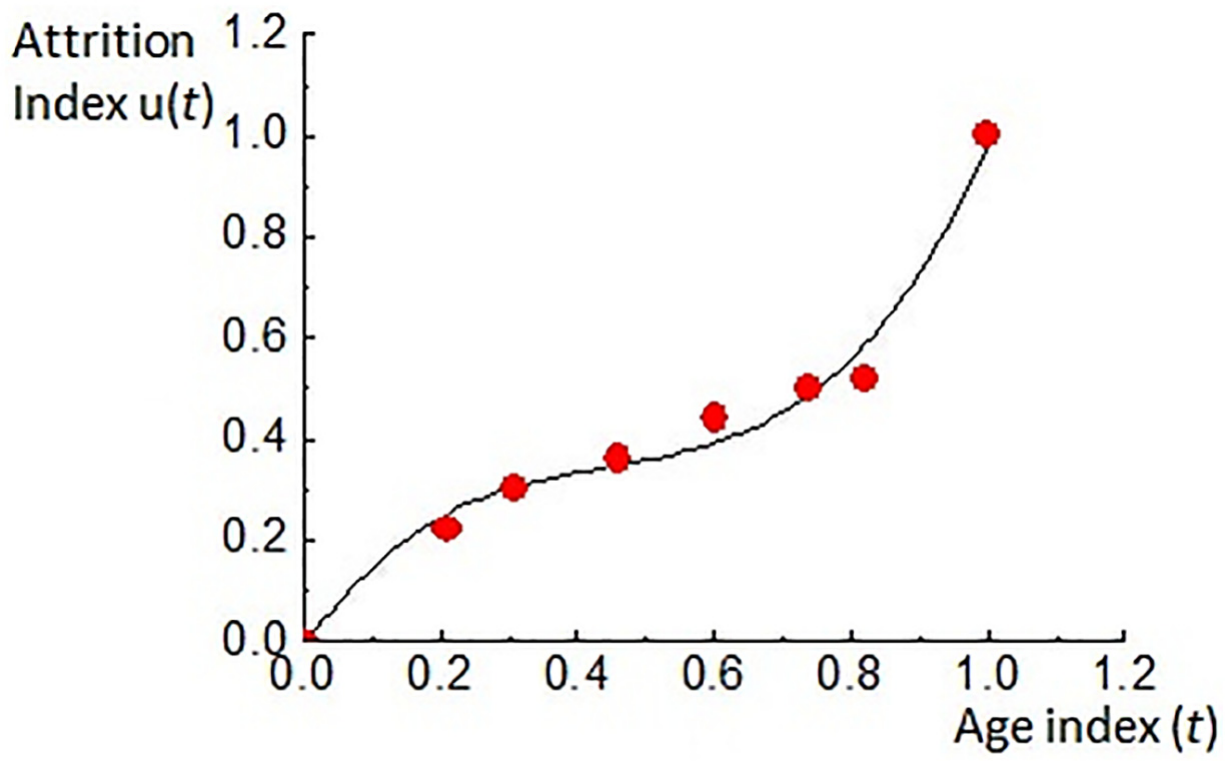
Dynamic distribution curve of attrition.

Data in Table 2 are normalized with formula (12). In Table 3, *X*_*i*_ and *Y*_*i*_ respectively represent the age and attrition indices after *x* and *y* are calculated with the min-max normalization. Then, the data are fitted by adopting a cubic polynomial whose analysis formula is *u*(*t*) = 2.899*t* ^3^−3.849*t* ^2^+1.94*t*−0.013 by Matlab software and the dynamic distribution curve of attrition is shown in Fig 6

**Table 3:** Average Attrition Index of lower molar after normalization.

**Age *X*_*i*_(year)**	0	0.21	0.31	0.46	0.6	0.74	0.82	1.0
**Attrition Index *Y_i_***	0	0.22	0.3	0.36	0.44	0.5	0.52	1.0

$$X=\frac{x-MinValue}{MaxValue-MinValue}$$ (12)

#### 3.2.3 Simulation experiment of nonlinear attrition

**Fig 7:**
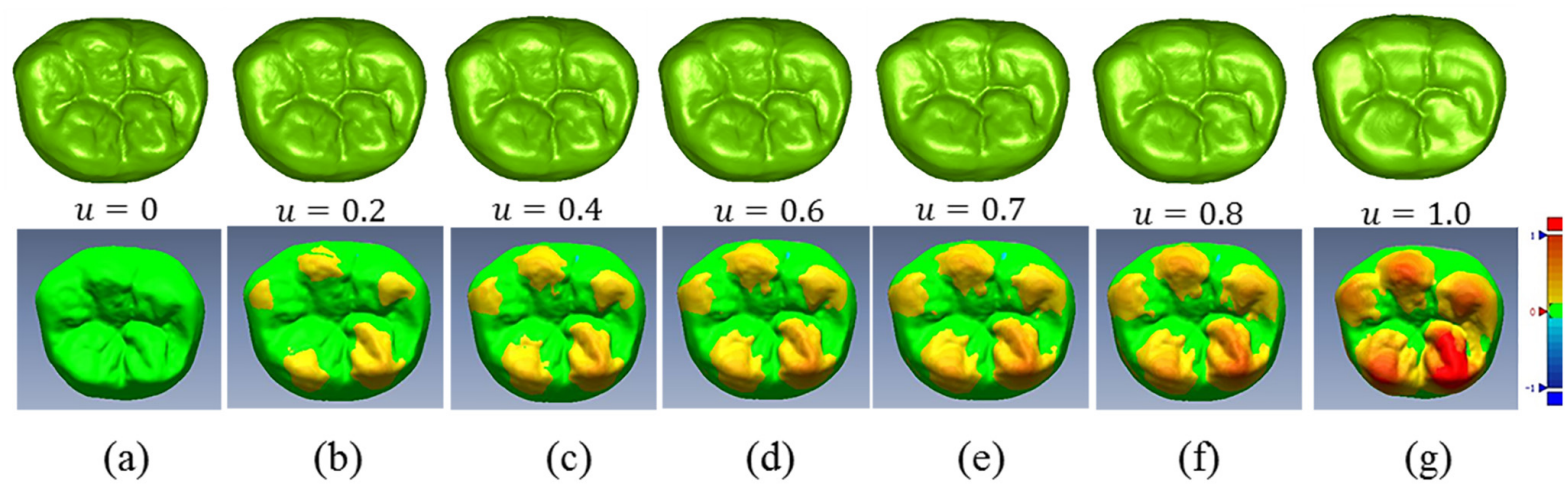
Non-linear attrition process simulation for the mandibular first molar attrition. a. tooth surface before attrition; b-f. tooth wear surface with parameter *u* from 0.2 to 0.8; g. tooth wear surface with parameter *u* = 1.0. The color from yellow to red shows the distribution area and degree of wear.

The attrition distribution curve (Fig 6) can be divided into three sections, 0–0.25, 0.25–0.7, and 0.7–1.0, with corresponding age phases 8–25, 26–55, and 56–78, exactly corresponding with the three phases of tooth wear (referring to the physiologic attrition of a permanent molar). During the early occlusion phase after tooth eruption, the enamel of a molar is immature and has a low degree of mineralization. It is a non-enamel rod layer with low surface hardness. Therefore, attrition is relatively fast. When entering the medium phase, the enamel rod in the dental enamel is aligned tightly, with full mineralization and maximal surface hardness. Wear resistance is at its best condition and attrition is minimal and stable. The gnarled enamel at the bottom of enamel layer is exposed during the later phase. Because the enamel rod is aligned sparsely, the average hardness is lower than before. When the wear is extended to the dentin layer, wear resistance is failing, surface striping and scratches are intensified and the attrition index increases rapidly. This attrition process is supported by clinical evidence [29]. Fig 7 shows a nonlinear simulation sequence of tooth wear and the distribution and color gradation strength of nonlinear simulation attrition.

## Discussion

This paper presents a method that uses 3D dynamic simulation of tooth wear morphology, which can effectively simulate the overall process of attrition (Fig 6). Compared with the tooth attrition levels defined by Smith [5], important anatomical features such as the initial cusp will gradually disappear over time. Tooth erosion on the occlusal surface during the morphing process is similar to tooth wear at each level in the 8-level classification. In addition, the morphing process is smooth and stable, without generating an abnormal intermediate state. Therefore, the method of image measurement proposed by Hove et al. [6] and Teaford et al. [7] can only be used for 2D measurement of partial parameters in attrition, and cannot analyze the 3D distribution area of tooth wear. Both Zou et al. [11] and Rodrigueza et al. [12] proposed a method aligning 3D public area to quantitatively compare and analyze the distribution and variation of the attrition area before and after tooth wear. However, no previous work has mentioned the method of modeling the attrition process. This study presents a novel method that allows dynamic reconstruction and simulation of the attrition process, thus improving the capabilities of 3D attrition analysis.

The process of attrition is complex due to its nonlinearity. Therefore, this article proposes a method using data of tooth wear from a certain population in a certain geographic region to extract an attrition dynamic control curve *u*(*t*) (Fig 6) for nonlinear attrition simulation. The nonlinear simulation sequence of attrition constrained by this DCF is shown in Fig 7. Fig 7(a) and 7(b) show the early occlusion phase; Fig 7(c)–7(e) show the medium occlusion phase with stable attrition; and Fig 7(f) and 7(g) show the later attrition phase with accelerated attrition. These images show that the proposed method can efficiently and dynamically simulate the nonlinear morphing process of the three phases of physiological attrition of permanent teeth. The lower portion of Fig 7(a)–7(g) shows the distribution area and strength of nonlinear attrition under the influence of DCF. By comparing Fig 7(d)–7(f) with Fig 5(d)–5(f), a significant influence can be seen on the distribution and strength of attrition, as well as the factual representation of the attrition process. As a result, the proposed method is an efficient tool for further analysis and prediction of attrition.

## Conclusions

1. Studying tooth wear is a very time-consuming process. This paper presents a novel method of tooth wear morphology simulation: first, it establishes feature alignment of the occlusal surface before and after attrition by using the tooth public area without attrition; second, it constructs a homogeneous attrition surface; finally, it reconstructs the sequential process of tooth wear based on Laplacian coordinates. This method can effectively simulate the entire attrition process with high efficiency and robustness.
2. Differences in populations, regions, food, and climate may lead to specific nonlinear features in tooth attrition. This paper proposed a method to transform the statistical distribution data of an average attrition level and age in a certain region into an attrition curve. With this attrition curve, the nonlinear process simulation of tooth wear can be achieved in a certain region.
3. With advancements in oral scanning technology, it is becoming easier to effectively collect digital dental models of patients. By using tooth wear digital simulation technology, the study and analysis of the process of tooth wear can be convenient, precise, and inexpensive. It can also provide a reliable means for exploring the attrition mechanisms of teeth.
4. Experimental evidence for the abnormal attrition of teeth can also be provided by the methods shown in this paper. Based on the attrition features of various teeth at different ages, it is possible to analyze the occlusal factors of occlusal disease and bruxism based on their occurrence, thus offering significant guidance for clinical therapies.
